# Highly Heterogeneous Probiotic *Lactobacillus* Species in Healthy Iranians with Low Functional Activities

**DOI:** 10.1371/journal.pone.0144467

**Published:** 2015-12-08

**Authors:** Mahdi Rohani, Nasrin Noohi, Malihe Talebi, Mohammad Katouli, Mohammad R. Pourshafie

**Affiliations:** 1 Department of Microbiology, Pasteur Institute of Iran, Tehran, Iran; 2 Department of Microbiology, School of Medicine, Iran University of Medical Sciences, Tehran, Iran; 3 Genecology Research Centre, Faculty of Science, Health, Education and Engineering, University of the Sunshine Coast, Maroochydore DC, Queensland, Australia; University of Ulm, GERMANY

## Abstract

**Background:**

Lactic acid bacteria (LAB) have been considered as potentially probiotic organisms due to their potential human health properties. This study aimed to evaluate both in vitro and in vivo, the potential probiotic properties of *Lactobacillus* species isolated from fecal samples of healthy humans in Iran.

**Methods and Results:**

A total of 470 LAB were initially isolated from 53 healthy individual and characterized to species level. Of these, 88 (86%) were *Lactobacillus* species. Biochemical and genetic fingerprinting with Phene-Plate system (PhP-LB) and RAPD-PCR showed that the isolates were highly diverse consisted of 67(76.1%) and 75 (85.2%) single types (STs) and a diversity indices of 0.994 and 0.997, respectively. These strains were tested for production of adhesion to Caco-2 cells, antibacterial activity, production of B12, anti-proliferative effect and interleukin-8 induction on gut epithelial cell lines and antibiotic resistance against 9 commonly used antibiotics. Strains showing the characteristics consistent with probiotic strains, were further tested for their anti-inflammatory effect in mouse colitis model. Only one *L*. *brevis*; one *L*. *rhamnosus* and two *L*. *plantarum* were shown to have significant probiotic properties. These strains showed shortening the length of colon compared to dextran sulfate sodium and disease activity index (DAI) was also significantly reduced in mouse.

**Conclusion:**

Low number of LAB with potential probiotic activity as well as high diversity of lactobacilli species was evident in Iranian population. It also suggest that specific strains of *L*. *plantarum*, *L*. *brevis* and *L*. *rhamnosus* with anti-inflammatory effect in mouse model of colitis could be used as a potential probiotic candidate in inflammatory bowel disease to decrease the disease activity index.

## Introduction

The concept of using live bacterial species such as *Lactobacillus spp*. with health benefit has received a great deal of attention in recent years. It is well known that the gastrointestinal (GI) tract is the home to a vast number of bacterial species, with vital roles in maintaining GI functionality including up to 70% of the immune system activity [1]. The probiotics are recommended as a preventive approach to maintain the balance of intestinal microbiota [2]. Amongst various microbiota, *Lactobacillus sp*. is especially important for the maintenance of the human intestinal microbial ecosystem [3] which, in turn, may affect the quality of life. It has been indicated that the disturbances in the normal microbiota of the GI tract may lead to dysbiosis and ultimately clinical disease expression [4].

Probiotics have been used to modulate immunity, reduction of cholesterol, symptoms of crohn’s disease, atopic dermatitis, diarrhea and constipation duration and also to treat disease conditions such as candidiasis, urinary tract infections and rheumatoid arthritis [5].

In recent years, various studies have shown that resident bacterial flora play a central role in pathogenesis of inflammatory bowel diseases (IBD) [6], partly, due to immunomodulatory properties of probiotic organisms [7] indicating a their role in prevention and/or therapy of IBD cases [8, 9]

Isolation of probiotic bacteria and determination of their biological activities has been under intensive investigations in developed countries. However, less attention has been given in developing or under-developed countries where the idea of the probiotic is still in its infancy. In this study we aimed to isolate and characterize *Lactobacillus* species with probiotic properties from fecal samples of healthy populations in Iran with special focus on their efficacy in an animal model of IBD.

## Materials and Methods

### Experimental Design

Fecal samples from 53 volunteers healthy individuals aged between 1 and 36 years old were collected. The volunteers were of different background living in Tehran City, Iran (n = 32) and or north part of Iran (n = 21). Criteria for selection of volunteers included lack of any antibiotic therapy over 6 months pre-sampling, did not have any significant GI disorders or consumed any probiotic products.

Fecal samples were serially dilutions in PBS (pH = 7.4)), plated onto Man, Rogosa and Sharpe (MRS) agar (Merck, Germany) and incubated at 37°C for 48h in an anaerobic condition. From each sample 10 isolated colonies (where possible) was selected, further tested for purification and kept at -80°C in MRS broth with 20% glycerol until tested.

### pH and bile tolerance test

All LAB strains were initially tested for their ability to resist to bile salts and pH as described before [10]. In brief, bacterial cells were grown overnight in MRS broth after which the culture was centrifuged at 6000rpm and the pellets were washed in PBS. An initial count (C_0_) of the bacterial suspension was performed after serial dilution (10^−2^ to 10^−10^)and plating on MRS agar followed by incubating plates anaerobically at 37°C for 48h. Then bacterial pellets re-suspended in 5ml PBS and the pH was adjusted to 3.0 using HCl (Merck, Germany) and incubated at 37°C for 3h. To determine the bile resistance property of the isolates, bacterial pellets, prepared as described above, were re-suspended in MRS broth containing 0.4% bile salts (Merck, Germany) and incubated for 6h. Harvested cells from the PBS at pH 3.0 were washed in PBS and the enumeration of bacteria cells was performed as described above. Viable cells were grouped as strongly resistant, resistant, intermediate and susceptible based on 2, 2–4, 4–6 and >6 log reduction in comparison with the initial suspension after 3 and 6h of incubation in acid and bile respectively.

### Genus and species identification of *Lactobacillus sp*.

Total DNA was extracted using peqGOLD Bacterial DNA Kit (peQlab, Germany) according to the manufacturer's instruction. Molecular identification of *Lactobacillus* genus was carried out by PCR using the previously designed *Lactobacillus* 16s rRNA specific primers for-lac (5´-TGGAAACAGGTGCTAATACCG-3´) and Rev-lac (5´-CCATTGTGGAAGATTCCC-3´) [11]. The PCR was performed under the following conditions: 94°C for 5 minutes; 30 cycles of 94°C for 30 seconds, 57°C for 30 seconds and 72°C for 30 seconds, and a final extension step at 72°C for 7 minutes for all 102 acid and bile resistant LAB isolates.

For species identification of isolated *Lactobacillus* sp., multiplex PCR containing species-specific primers for *L*. *acidophilus*, *L*. *casei-group*, *L*. *delbrueckii*, *L*. *gasseri*, *L*. *plantarum*, *L*. *rhamnosus* and *L*. *reuteri* were used using the following conditions: after one cycle at 94°C for 5 min, 40 cycles of 94°C for 30 seconds, 51°C for 40 seconds and 72°C for 30 seconds were performed. Final extension was carried out at 72°C for 7 minutes [12].

### Phenotypic classification with Phene Plate system (PhPlate)

All acid and bile resistance lactobacilli isolates (n = 88) were subjected to a biochemical fingerprinting with the PhPlate system according to the manufacturer’s instructions (PhPlate Micro-plate Techniques AB, Sweden) modified for lactobacilli (PhP-LB)typing. The microplates (PhP-LB) contain 4 sets of dehydrated reagents (23 different sugar including arabinose, xylose, galactose, maltose, cellobiose, trehalose, palatinose, sucrose, lactose, melibiose, mannose, melezitose, inosin, mannitol, arbutin, sorbitol, gallactose, sorbose, rhamnose, taghatose, amygdalin, gluconate, salicin) which have been specifically selected for typing of *Lactobacilli*. PhP-LB plates were incubated at 37°C and bacterial utilization of the substrates of each well was measured after 24, 48, and 72h by scanning the images. Scanned images were analyzed by software package PhPWIN (PhPlate Micro-plate Techniques AB, Sweden). Mean similarity between duplicate assays of all strains -2 SD was calculated as identification level (ID) which was 0.975. Strains with similarity more than 0.975 were regarded as identical and grouped to the same PhP type.

### Genotypic classification with RAPD-PCR

All 88 acid and bile resistance lactobacilli isolates were also subjected to Random Amplified Polymorphic DNA (RAPD) using previously designed oligonucleotide OPL-05 and PL-1 with some modifications [13]. PCR reaction for each primer was done in a separate tube and run in the same well in 1.5% agarose gel for more discrimination. The PCR was performed with the following conditions: 94°C for 2 minutes; 40 cycles of 94°C for 30 seconds, 37°C for 30 seconds and 72°C for 2 minutes with a final extension step at 72°C for 10 minutes. Strains were clustered with the UPGMA method using the software Gel compare II version 4.0 (Applied Maths, Sint-Martens-Latem, Belgium).

### B12 production assay

All 88 isolated lactobacilli were tested for production of B12 vitamin based on competitive binding radio assay using commercial kit Simultrac-SNB (MP Biomedicals, USA). Briefly, all acid and bile resistance *Lactobacillus* strains were inoculated in MRS broth and incubated anaerobically in 37°C for 96h. Supernatants were then collected after centrifugation and stored at −80°C before assayed. The pellet radioactivity of each sample was counted using a gamma counter (Laboratory Technologies Inc., USA). MRS broth medium without bacteria was used as negative control.

### Antibiotic susceptibility testing

The agar disc diffusion method was used to determine the antibiotic susceptibility patterns of all acid and bile resistant lactobacilli isolates on MRS agar plate. Nine antibiotics including penicillin G (10μg), gentamicin (120μg), erythromycin (15μg), tetracycline (30μg), amoxicillin (25μg), ciprofloxacin (5μg), chloramphenicol (30μg), oxacillin (1μg), and streptomycin (10μg) (MAST Diagnostics, U.K) were tested. The discs were placed on the agar plates, and incubated at 37°C for 48h. The diameters of inhibition zones were measured and the results were expressed as sensitive and resistant according to clinical and laboratory standards institute (CLSI) guideline [14]. Because of the importance of resistance transfer in gut, resistant strains to gentamicin, erythromycin, tetracycline and chloramphenicol were excluded from other selections. Minimum inhibitory concentration (MIC) of ampicillin, vancomycin, gentamicin, kanamycin, streptomycin, erythromycin, clindamycin and tetracycline was also conducted according to Klare et al. [15].

### Attachment to Caco-2 cells

The adherence of acid and bile resistance lactobacilli to Caco-2 cells was examined as previously described by Jacobsen et al. [3]. Caco-2 cells were cultured in a monolayer with RPMI (Gibco, Carlsbad, CA, USA) with 20% (v/v) fetal calf serum (Gibco, Life Technology, USA) and antibiotics (100U. ml^-1^ penicillin, 100mg.ml^-1^ streptomycin) at 37°C in a 5% CO_2_ atmosphere. Initially, 3ml of Caco-2 cells with 1.5×10^5^cells/ml were seeded on 6-well cell culture plates and after confluency the cells were washed twice with 3ml PBS. An aliquot of two ml of RPMI (without antibiotics) was added to each well and incubated at37°C for 3h. Bacterial cultures (10^9^cfu/ml) were suspended in 1ml RPMI1640 medium (without antibiotics) and then added to different wells. The plates were incubated at 37°C in 5% CO_2_ for 1h. After incubation, all of the wells were washed four times with PBS to release unbound bacteria and filled with 1 ml methanol and incubated for 5–10min at room temperature to fix cells and attached bacteria. Staining was made with 3 ml of Giemsa stain solution (1:20) (Sigma-Aldrich Co., Mo, USA) and incubated 30min at room temperature. Excess stain was removed with distilled water, dried and examined under oil immersion microscope. The adherent lactobacilli in 20 random microscopic fields were counted for each test and cells showing between 0 to 40 attached bacteria in 20 fields were regarded as non-adhesive, while 41 to 100 attached bacteria were regarded as an adhesive and >101 attached bacteria were regarded as strongly adhesive.

### Determination of antibacterial activity

Based on data obtained from other screening tests (bile and acid resistance, attachment to Caco-2 cells, antibiotic resistance pattern and B12 production), 20 *Lactobacillus spp*. were selected and tested for their antimicrobial activity using an agar diffusion method as described by Fernandez et al [16]. Suspensions of approximately 10^8^cells/ml of pathogens including *Shigella soneii* (ATCC 12022), *Pesudomonas aeruginosa* (ATCC 27853), wild types of *E*. *coli* strains belonging to three pathotypes i.e. enteropathogenic *E*. *coli* (EPEC) (ATCC 43887), enterotoxigenic *E*. *coli* (ETEC) and enteroaggregative *E*. *coli* (EAEC) (ETEC and EAEC were from our culture collection center at the Pasteur Institute of Iran and were not type strains) [17, 18], *Salmonella typhi* (ATCC 19430), *Proteus mirabilis* (ATCC 25933), *Yersinia enterocolitica* (ATCC 23715), *Streptococcus agalactiea* (ATCC 12386) and *Listeria monocytogenes* (ATCC 19113) were poured on plates containing Muller Hinton agar, except for *L*. *monocytogenes* that was onto BHI agar. Plates were then punched with 6 mm diameter bores and each well was filled with 100μl of the culture of selected *Lactobacillus* strains grown in MRS broth overnight. The plates were then incubated at 37°C under aerobic condition for assessing the antimicrobial effect of each *Lactobacillus* isolates. After 24h of incubation, one millimeter or greater of inhibition zone were scored as positive antibacterial activity.

### Anti-proliferation effect of LAB on Caco-2 cells

This was monitored using XTT [2,3-Bis-(2-Methoxy-4-Nitro-5-Sulfophenyl)-2H-Tetrazolium-5-Carboxanilide] assay for the 20 selected strains as previously described [19]. Caco-2 cells were seeded at a concentration of 2×10^5^ cells/mL in 96-well plates with 250μl of fresh RPMI1640 medium supplemented with glutamine 1% (w/v) and fetal calf serum [10% (v/v)] in each well. Wells were then inoculated with *Lactobacillus sp*. isolates, at a multiplicity of infection (MOI) of 100 alone. Microplates were incubated at 37°C under 5% CO_2_. After 16h, cell viability was checked by trypan blue, supernatants were removed and cell monolayers were washed twice in PBS. Then wells we refilled with 100μl of RMPI medium without phenol red and antibiotic and 50μl of XTT kit reagents (Roche, Germany) was added to each well and plates were incubated at 37°C in 5% CO_2_. After 6h, formazan absorbance was assayed at 450nm. The percentage of proliferation was calculated as follows: OD _positive_−OD _negative_ / OD _negative_ × 100 where optical density positive (OD_positive_) and negative (OD_negative_) represent Caco-2 cells treated with potentially probiotics and Caco-2 cells alone, respectively. The test was done in triplicates.

### Interleukin-8 induction

For determination of putative influence of 10 final selected strains in IL-8 production [19], HT-29 cells were seeded at a concentration of 2×10^5^ cells/ml in 24-well plates and cultured for 7 days at 37°C and 5% CO2 to reach confluence. The cell monolayer’s were washed twice with PBS and then 250μl of fresh RPMI1640 medium supplemented with glutamine 1% (w/v) and fetal calf serum [10% (v/v)]without antibiotics were added to each well. Monolayers were treated by LPS (Sigma, Saint Louis, USA) at 100ng/ml and then bacterial culture were suspended in 1 ml RPMI 1640 medium (without antibiotics) and then added to different wells at a MOI of 100. For evaluation of absence of our selected *Lactobacillus sp*. non-activated HT-29 cells were treated with our selected bacteria. After 6h of incubation at 37°C and 5% CO_2_, cell viability was checked and supernatants were stored at −80°C before being assayed. Quantification of secreted IL-8 in all supernatants was done by ELISA test (eBioscience, Inc., San Diego, USA) according to manufacture instruction.

The percentage of secreted IL-8 variation was calculated as follows:

$${\text{Value}}_{\;\text{assay}}-{\text{Value}}_{\;\text{negative control}}/\;{\text{Value}}_{\;\text{positive control}}-{\text{Value}}_{\;\text{negative control}}\;\times \;100$$

Where positive and negative controls represent HT-29 cells treated by LPS and HT-29 cells alone without treatment, respectively. The test was done in triplicates.

### Anti-inflammatory effect of selected strains in mouse colitis model

The anti-inflammatory effect of the selected *Lactobacillus* strains was assessed in a chemically induced inflammatory bowel disease (IBD) with dextran sulfate sodium (DSS) in Balb/c mice as described before [20]. Balb/c mice (n = 60) were placed in groups of 5. Ten groups were gavage with 0.5 ml PBS containing *Lactobacillus spp*. at the concentration of 10^9^three days before IBD induction and continued up to 7 days. After three days, water was replaced with DSS solution in all cages except for the control group. All other groups that consumed DSS solution were gavage with *Lactobacillus spp*. until day seven except for DSS control group. Groups 3 to 12 were gavage with selected *Lactobacillus spp*. which included strains of *L*. *plantarum 42*, *L*. *plantarum 156*, *L*. *brevis 165*, *L*. *rhamnosus 195*, *L*. *brevis205*, *L*. *plantarum 240*, *L*. *plantarum 241*, *L*. *plantarum 365*, *L*. *plantarum 467* and *L*. *brevis 470*. After day 10, all mice were scarified and middle sections of colons were kept in 1.5 ml micro tube at -70°C until evaluation of myeloperoxidase (MPO) activity [20]. In this study three different features in the gut of mice were compared between DSS group and other 11 groups for selection of most effective isolates in preventing IBD induction in *in vivo* model. These three features included colon length, disease activity index (weight loss, stool consistency and occult/gross bleeding) and MPO activity in the middle section of colon tissue [20, 21].

### Statistical analysis

Statistical analysis was carried out using SPSS (version 16, SPSS Inc., Chicago, IL) software package. The correlation between isolated species and age, birth place and gender of the volunteers with all biological activities were tested using non-parametric one-way ANOVA (Kruskal-wallis) and followed by Fisher’s least significant difference (LSD) for post hoc tests. Differences of P < 0.05 were considered statistically significant.

## Results and Discussion

### Isolation and identification of *Lactobacillus* species

Out of 470 LAB isolated and tested, 102 isolates were able to resist low pH (i.e. pH 3.00) and 0.4% bile salts which belonged to *Enterococcus*, *Pediococcus* and *Lactobacillus* genus. Among these isolates88 (18.7%) were identified as *Lactobacillus* species. Results indicated that the majority of our isolates did not survive transiently through the GI tract. Obviously, other bacteria such as viable but nonculturable (VBNC), which might have been present in our samples, were not picked-up for the experimental analysis. Additionally, anaerobic condition, used in this study for the screening tests, might have corresponded to the close colonic milieu for the isolation of *Lactobacillus*. On the other hand, MRS with pH of around 5.5 was used as the culture medium for the determination of certain biological activities such as vitamin B12 production by *Lactobacillu*s. This medium, therefore, may not be an actual representative of the pH that exists in the colon.

**Fig 1:**
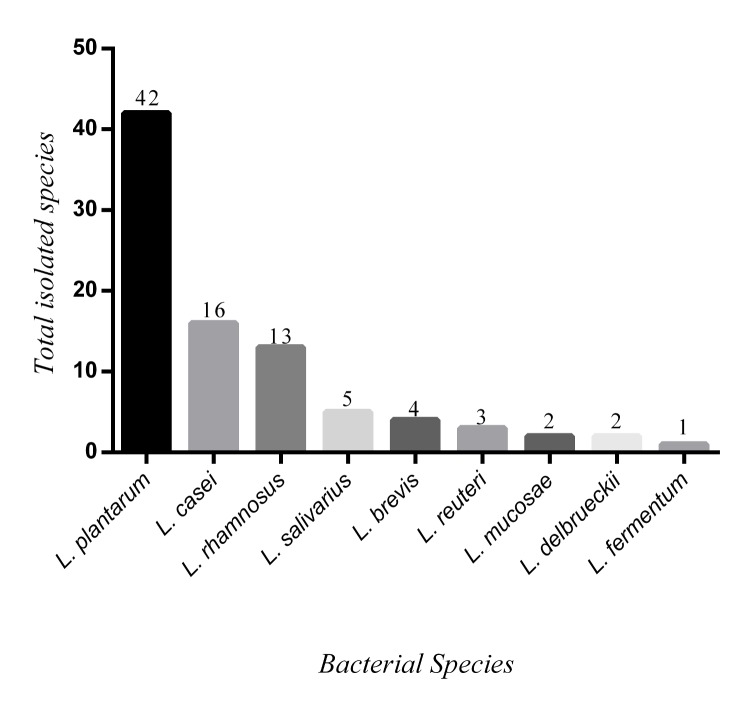
Determination of *Lactobacillus* species that have resistant to acid (pH 3) and bile salts.

The 88 strains were then identified by multiplex PCR and sequencing to 9 species (Fig 1).Of the total isolates, a significant numbers of lactobacilli did not show any probiotic properties and eventually were excluded from the study. Upon the final selection, the isolates which showed significant properties in biological tests were independent from the age of the donors and no correlation could be described (p = 0.018), suggesting lack of age-probiotic associated relationship.

**Fig 2:**
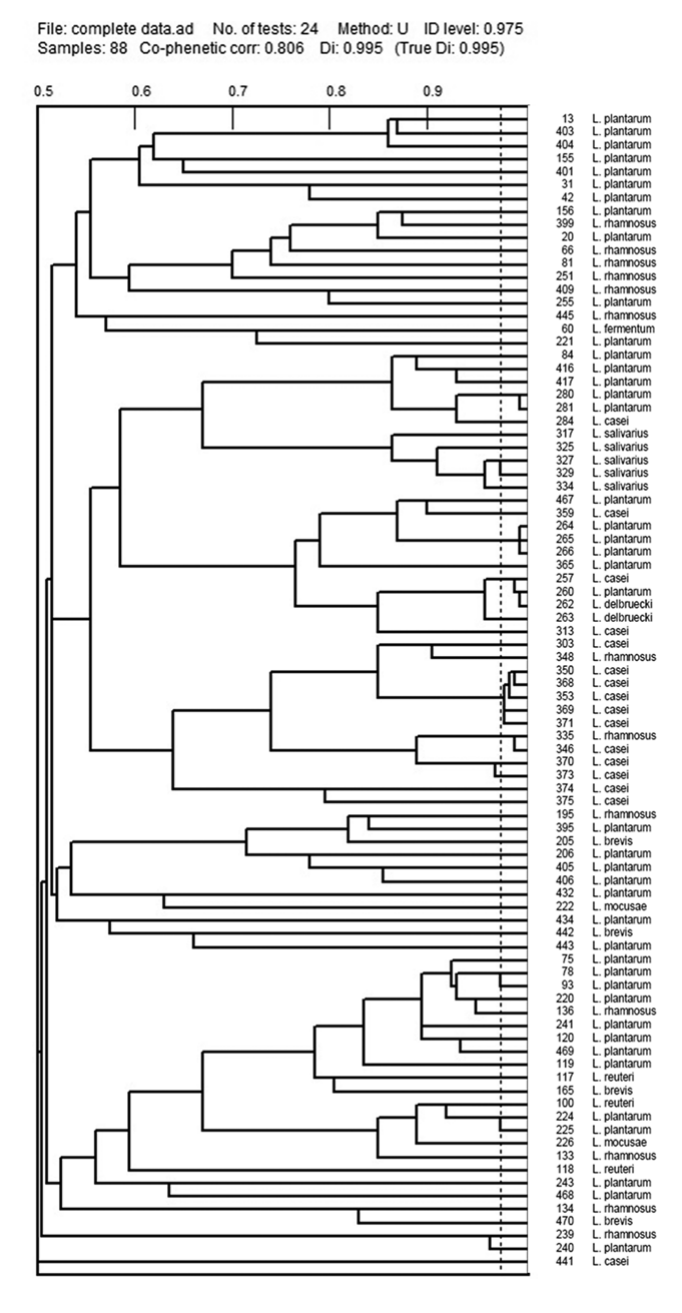
Phenotypic clustering of isolated *Lactobacillus sp*. with PHP-plate assay.

**Fig 3:**
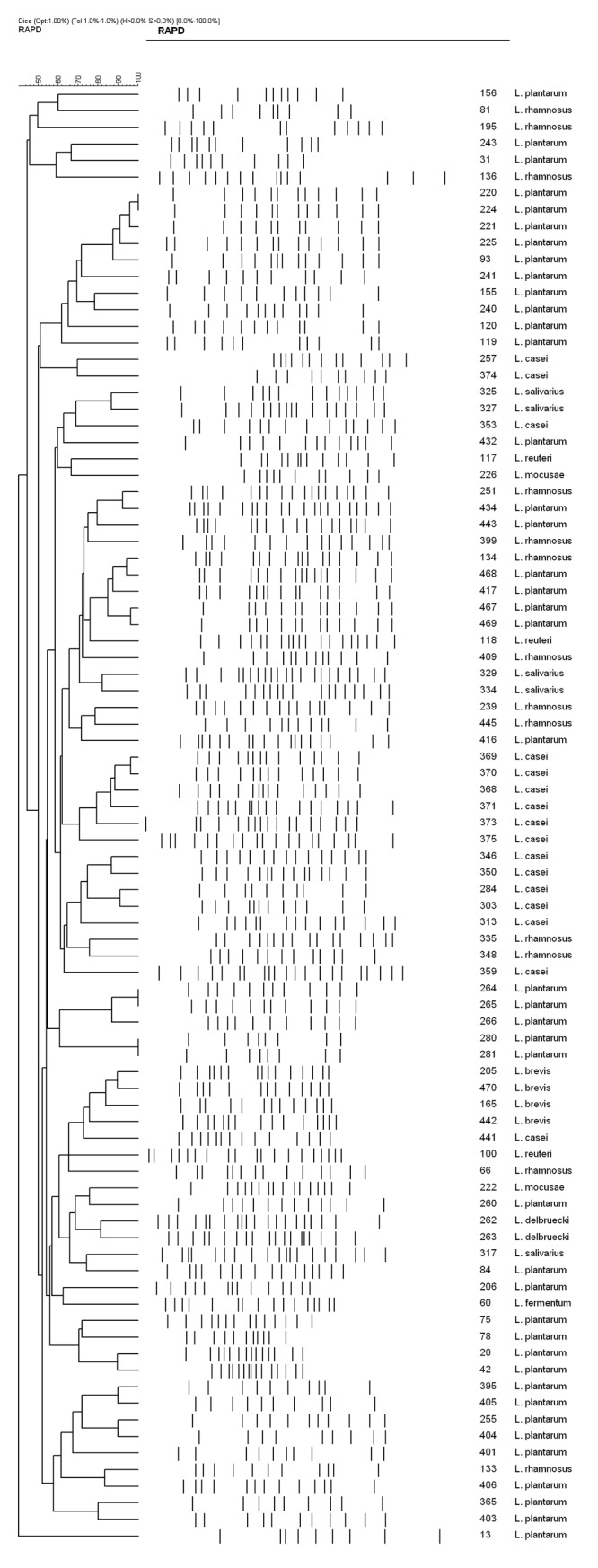
Genotypic clustering of isolated *Lactobacillus sp*. with RAPD PCR assay.

The *Lactobacillus* isolates were phenotyped using Phene Plate system (PhP-LB) (Fig 2) and genotyped by Random Amplified Polymorphic DNA (RAPD-PCR) (Fig 3). PhP-LB results showed the presence of 8 common types (CTs) and 67 single types (STs). RAPD-PCR resulted in 6 CTs with 13 isolates (14.8%) and 75 (85.2%) STs. The diversity of the isolates using Simpson’s index showed a high diversity among the isolates with both methods i.e. PhP-LB, Di of 0.994 and RAPD-PCR Di of 0.997. Combination of the two techniques resulted in Di = 0.999 with 84 STs. The results from PhP-LB indicated that the combination of the two techniques could result in a significant discriminatory power than each of the techniques used alone. In addition, the results showed that the *Lactobacillus* species isolated from Iranian population is highly heterogeneous with no specific disseminated clones.

### Vitamin B12 production

**Fig 4:**
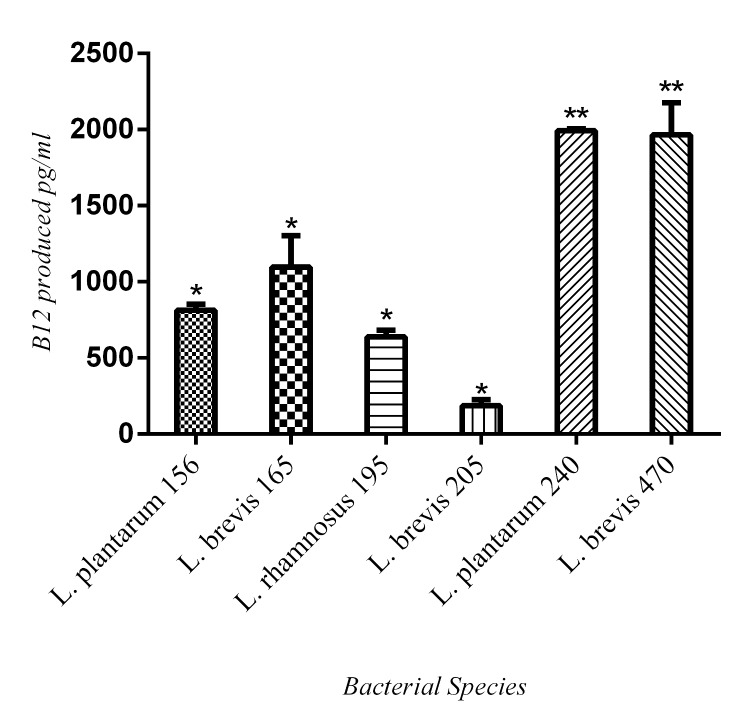
B12 vitamin production by 6 different *Lactobacillus sp*. The results are reported as the mean ± SD of triplicate samples. Arrow indicates standard deviation and means comparison was done with unpaired t-test. * indicates statistically significant production of vitamin B12 by the isolates in comparison with the negative control samples (p<0.05). ** indicates highly significant vitamin B12 production in comparison with other 4 positive isolates (p<0.001).

Six out of the 88 isolates (7%) i.e. *L*. *plantarum* (2), *L*. *rhamnosus* (1) and *L*. *brevis* (3) were identified as vitamin B12 producers (Fig 4). Significant vitamin B12 production (>2000 pg/ml) was observed in one *L*. *plantarum* and one *L*. *brevis* strains. These 6 strains were isolated from 4 individuals.

Endogenous lactobacilli are important vitamin supplier in humans. World Health Organization (WHO) has recommended that a level of <150 pmol/L (<203 pg/mL) is considered as vitamin B12 deficiency [22]. Although lactobacilli flora isolated from volunteer population do not represent the entire flora of the Iranian populations, it does coincide with a multicenter study where 29% of the Iranian population were found to be vitamin B12 deficient as compared to the populations from 8 other countries [23].

### Antibiotic resistance pattern

Antibiotic susceptibility tests showed that all of the strains were sensitive to chloramphenicol, amoxicillin and penicillin G. Different degrees of resistant to ciprofloxacin (100%), oxacillin (86.4%), streptomycin (38.6%), tetracycline (36.4%) and erythromycin (8%) were observed. The antibiotic susceptibility test and minimum inhibitory concentration (MIC) of the isolates showed that their resistance patterns were within the acceptable range as suggested by the EFSA guideline for the probiotics [24]. The isolates that had high resistance level to each of the tested antibiotics and carried one or more plasmids (data not shown) excluded from further evaluations (S1 Fig).

### Attachment to Caco-2 cells

Different level of adhesion to Caco-2 was observed among the isolates. In all, 44 (50%) isolates were non-adhesive (i.e. with fewer than 40 bacteria attached in 20 microscopic fields), 19 (22%) were adhesive (with 41–100 bacteria) and 25 (28%) were considered strongly adhesive (>101 bacteria). There was no specific correlation between adhering to Caco-2 cells among different species of lactobacilli (p = 0.43).

Colonization capacity is an important criterion for probiotic strains [25] which, in turn, could stimulate immune system [1] and antagonized adhesion of enteropathogens [26]. In this study, 44 strains (50%) of *Lactobacillus* were identified as either adhesive or strongly adhesive as determined by *in vitro* attachment assay. Three strongest adhesive strains were *L*. *reuteri 100*, *L*. *rhamnosus 133* and *L*. *casei 313* with 491, 399 and 395 bacterial cells in 20 different microscopic fields, respectively. The adhesion index of our strains was significantly higher than *L*. *rhamnosus* GG adhesion index which has been reported by other investigators using similar protocol as ours by counting attached bacterial cells in 20 random microscopic fields [27].

### Antibacterial activity

Amongst the isolated strains, 20 *Lactobacillus* were found to have antibacterial activities against up to ten different species (Table 1). All examined *Lactobacillus* isolates inhibited the growth of EAEC. Amongst different strains of *E*. *coli*, less inhibition was observed against ETEC. Some strains such as *L*. *plantarum 13*, *L*. *plantarum156*, *L*. *plantarum78*, *L*. *plantarum220* and *L*. *rhamnosus136* inhibited all the tested pathogens. Out of 11 *L*. *plantarum*, 55% showed antibacterial activities against all the tested bacteria with the exception of *S*. *typhi*, a single *L*. *reuteri 118* was also active against all the other tested bacterial species. The results also showed that any *Lacto*ba*cillus* isolate could inhibit at least 2 entero-pathogenic bacteria.

**Table 1:** Antibacterial effect of total 20 selected *Lactobacillus Spp*. against pathogens bacteria.

Bacterial No.	*ShigellaSoneii*	*EPEC*	*EAEC*	*ETEC*	*Listeriamonocytogenes*	*Pseudomonasaeruginosa*	*Yersiniaenterocolitica*	*Salmonellatyphi*	*Proteus mirabilis*	*streptococcusagalactiea*
*L*. *plantarum 13*	+	+	+	+	+	+	+	+	+	+
*L*. *plantarum 42*	+	+	+	-	W	+	+	+	+	+
*L*. *plantarum 156*	+	+	+	+	+	+	+	+	+	+
*L*. *plantarum 467*	+	+	+	+	+	-	+	+	-	ND
*L*. *casei 359*	+	+	+	+	-	+	+	+	+	ND
*l*. *plantarum 365*	+	+	+	+	-	+	+	+	+	ND
*L*. *casei 313*	+	+	+	+	+	-	+	+	+	+
*L*. *rhamnosus 195*	-	+	+	-	-	-	-	+	-	-
*L*. *brevis 205*	+	+	+	-	+	-	-	+	+	+
*L*. *plantarum 75*	+	+	+	+	-	W	W	+	-	-
*L*. *plantarum 78*	+	+	+	+	+	+	+	+	+	+
*L*. *plantarum 220*	+	+	+	+	+	+	+	+	+	+
*L*. *rhamnosus 136*	+	+	+	+	+	+	+	+	+	+
*L*. *plantarum 241*	+	+	+	+	+	-	+	+	+	+
*L*. *brevis 165*	-	-	+	+	-	-	-	+	-	-
*L*. *plantarum 224*	+	+	+	+	+	-	W	+	+	+
*L*. *rhamnosus 134*	+	+	+	+	+	-	-	+	-	-
*L*. *brevis 470*	-	+	+	-	+	-	+	-	-	ND
*L*. *plantarum 240*	+	+	+	+	+	-	W	+	+	+
*L*. *reuteri 118*	+	+	+	+	+	+	+	-	+	+

+: Pathogen growth inhibited >1mm -: Pathogen growth not inhibited, W: weak pathogen growth inhibited ≤1mm, ND = not determined

Overall, the results from antibacterial activity showed that some of our isolates could produce substances which, in turn, inhibit the growth of gut pathogens. In a recent study, it was shown that amongst the Iranian the frequency of diarrhea caused by ETEC was significantly higher than EPEC and EAEC strains which might have been as the result of gut microbiota imbalance [28].The use of lactobacilli studied here might, therefore, be beneficial for populations who have diarrheal disease caused by ETEC. The least inhibitory activity by our Lactobacillus isolates was shown against *P*. *aeruginosa*. Almost 50% of our bacterial isolates did not show anti *P*. *aeruginosa* activity. It has been suggested by Tharmaraj et al. [29] that in vitro antibacterial effect of probiotics against *P*. *aeruginosa* in an aerobic condition is not a reliable evaluation setting.

### Anti-proliferation effect of *Lactobacillus sp*. on Caco-2 cells

**Fig 5:**
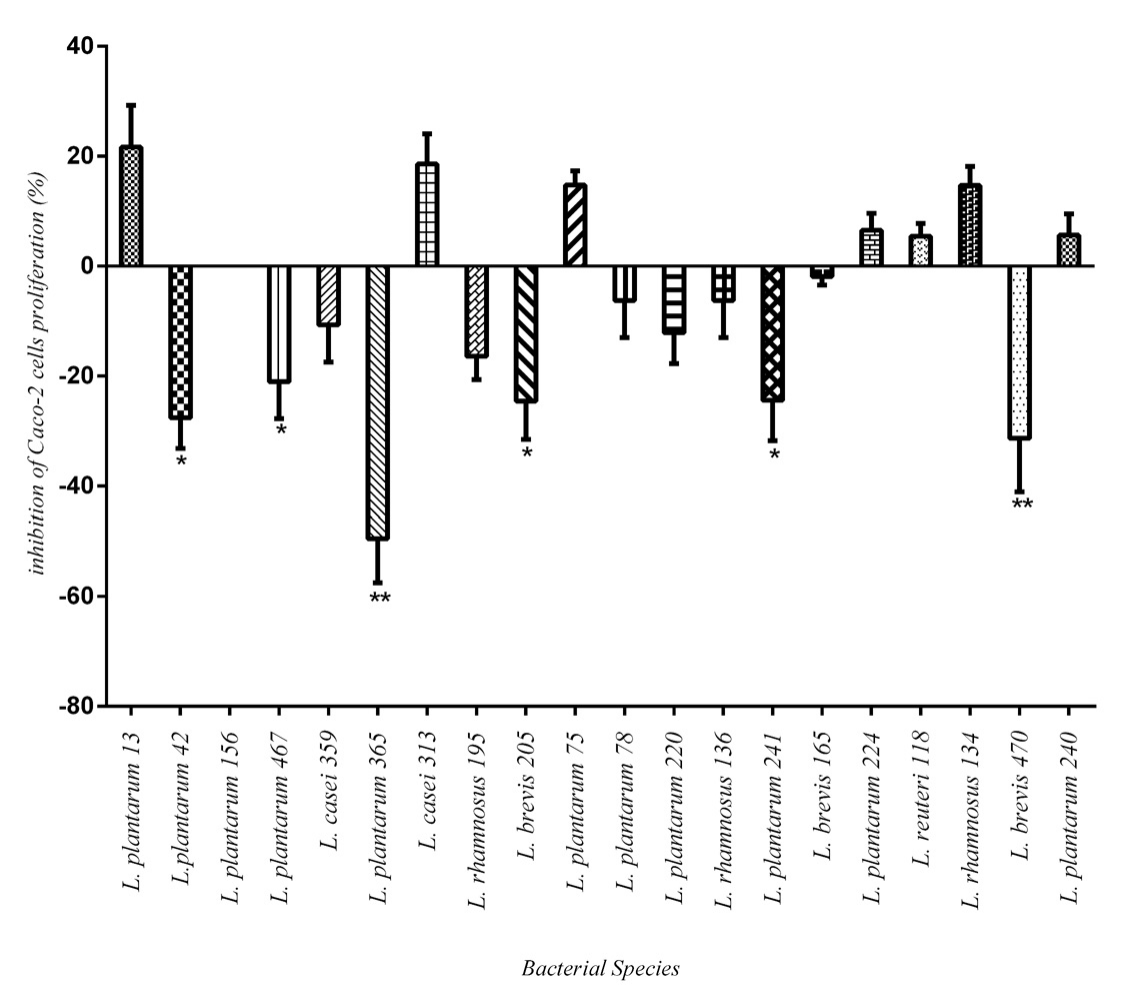
Anti proliferation effect of selected *Lactobacillus sp*. on Caco-2 cells using XTT. The results are reported as the mean ± SD of triplicate samples. The level of 20% inhibition of proliferation of Caco-2 was used as a criterion for anti-proliferative effect. Means comparison was done with unpaired t-test.* indicates significant anti proliferation effect by 6 *Lactobacillus sp*. (p<0.05). ** indicates highly significant anti proliferation effect (p<0.001).

Grimound et al. [19] have suggested a 20% inhibition of proliferation of Caco-2 cells could be used as a selection criterion for anti-proliferative effect. Amongst the 20 *Lactobacillus* strains tested, 6 strains (i.e. four *L*. *plantarum* and two *L*. *brevis*) showed decreased proliferation by more than 20% (Fig 5). The other 14 strains showed either less than 20% or no effect on the proliferation of Caco-2 cells after 16h of incubation. The most anti-proliferative effect was observed with *L*. *plantarum365*with an inhibition rate of 44.9±4.9%.

No correlation was found between specific species of *Lactobacillus* strains and anti-proliferation Caco-2 cell line (p = 0.316). The importance of *in vitro* anti-proliferation effect of probiotic bacteria is still unknown, although there are some hopeful prospects in their use in cancer prognosis [30] which supported by previous studies that have reported the changes in gut microbiota in colorectal cancers and adenoma [31,32]. In our study, *L*. *plantarum* strains *42*, *241*, *365 and 467* showed significant antibacterial and anti-proliferative activities. On the other hand, *L*. *plantarum* strains *13*, *156* and *78* showed significant antibacterial activity against all of the bacterial species examined with no anti proliferative activity. The comparative analysis of anti-proliferative and antibacterial effect by *Lactobacillus* isolates suggested that different and perhaps multiple biological factors might have been produced and/or exerted by these strains when present in the two different milieus.

### Anti-inflammatory effect of selected isolates in in vitro and in vivo models

**Fig 6:**
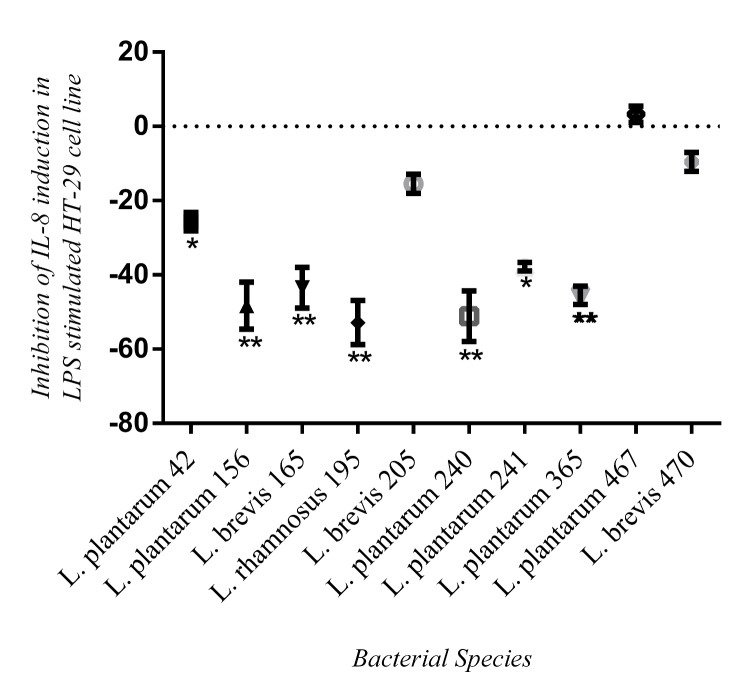
Inhibition of secreted IL-8 of selected *Lactobacillus* isolates. These isolates were selected based on their significant activities found in Figs 4 and 5. The results are reported as the mean ± SD of triplicate samples. Mean comparison was done with unpaired t-test. * & ** indicate statistically significant difference p<0.05 and p<0.001, respectively, for reduction of IL-8 production by lactobacilli isolates in HT-29 cell line stimulated with LPS.

None of the tested strains induced secretion of IL-8 in HT-29 cells by themselves (Fig 6). The results showed *L*. *rhamnosus 195*, *L*. *plantarum 240* and *L*. *plantarum 156* decreased LPS- induced IL-8 production by >50% (55.1, 54.8 and 50.6%, respectively). No significant difference in reduction of LPS-induced IL-8 was found amongst the different species of *Lactobacillus* (p = 0.0509).

In animal model used we scored disease activity index (DAI) based on weight loss, fecal character and fecal occult blood [20]. Administration of dextran sulfate sodium (DSS) shortened the colon length and increased DAI as expected. *L*. *plantarum* strains *42 and 156*, *L*. *rhamnosus* strain*195* and *L*. *brevis* strain *165* significantly reduced DAI score in the bacteria treated mice (Table 2). Administration of other *Lactobacillus* species had no effect on DAI of mice. The length of colon of mice in the DSS group was significantly decreased compared with the normal and *Lactobacillus* administrated groups except for the mice that were exposed to *L*. *plantarum 365* strain. All *Lactobacillus* strains could significantly (p<0.01) prevent the shortening of colon (Table 2).

**Table 2:** Effect of selected *Lactobacillus* sp. on myeloperoxidase (MPO) activity, disease activity index (DAI) and colon length, on DSS-induced colitis. Mean comparison was done with unpaired t-test. ** Highly significantly different from DSS group at P <0.01. *Significantly different from DSS group at P < 0.05.

Mice group	MPO activity (mU/mg)	DAI	Colon Length
*Control*	0.115±0.025**	0.1±0.01**	9.6±1.1**
*DSS*	0.236±0.023	1.7±0.4	7.1±1.1
*L*. *plantarum 42*	0.148±0.018**	0.1±0.2**	9.4±1.2**
*L*. *plantarum 156*	0.151±0.021*	0.3±0.5**	9.6±0.4**
*L*. *brevis 165*	0.123±0.032**	0.1±0.3**	10.2±0.6**
*L*. *rhamnosus 195*	0.142±0.028**	0.9±0.8*	10.3±0.3**
*L*. *brevis 205*	0.201±0.037	1.5±0.9	8.9±0.9**
*L*. *plantarum 240*	0.192±0.041	2.1±0.6	8.5±0.7*
*L*. *plantarum 241*	0.230±0.014	1.7±0.2	9.2±0.6**
*L*. *plantarum 365*	0.218±0.015	1.5±0.4	8.0±1.3
*L*. *plantarum 467*	0.161±0.027*	1.1±0.3	11.1±0.4**
*L*. *brevis 470*	0.169±0.031*	1.1±0.2	9.7±1.0**

As expected, a high activity of myeloperoxidase (MPO) in the DSS group was observed compared to the control group (P<0.001). The level of MPO activity was insignificant in the normal and the groups administrated with *L*. *plantarum* strains *42* and *156*, *L*. *brevis 165* and *L*. *rhamnosus 195*. In DSS and groups that were administrated with *L*. *plantarum* strains *240*, *241*and *365* and *L*. *brevis 205*, the MPO activity was highly significant (p<0.01) (Table 2).

In the present study, acute colitis was induced in Balb/c mice with oral administration of 2% DSS and the MPO activity was used to assess the tissue infiltration of neutrophils. Analysis of the level of MPO activity with DAI and colon length showed that DAI was directly related to the MPO activity where decreased DAI resulted in decreased MPO activity. Some reports [33, 34] have indicated that the reduced degradation of matrix in the intestinal mucosa could be related to the reduction in MPO activity. We, however, have found that the normal colon length alone with intact matrix, as determined in pathological examinations, had no correlation with MPO production, suggesting that other factor(s) may affect the outcome of MPO activity which, in turn, improves the disease state in potentially probiotic treated experimental model of colitis. IL-8 production by LPS-stimulated cells was inhibited by almost all *Lactobacillus* strains. The potentially probiotic strains which resulted in a significant reduction of MPO i.e. *L*. *plantarum* 42, *L*. *plantarum* 156, *L*. *brevis* 165 and *L*. *rhamnosus* 195 also showed significant (P <0.01) reduction in IL-8 induction as compared with DSS-induced group. In addition, MPO activity was unchanged when other *Lactobacillus* strains were administrated; suggesting that MPO activity in DSS-induced mice was not correlated with the IL-8 induction. Furthermore, the length of colon was reversed to its normal size, with exception of *L*. *plantarum*365, following potentially probiotic treatment of all DSS-induced animals. Our results also showed that only four of the selected strains i.e. *L*. *plantarum* strains *42 and 156*,*L*. *brevis 165* and *L*. *rhamnosus 195* could significantly reduce the IBD features including MPO activity, DAI and colon length as compared with DSS group.

## Conclusion

To our knowledge this is the first report on the isolation of LAB with probiotic characteristics from fecal samples of healthy human populations from different regions in Iran. The results suggest that some of the *Lactobacillus* strains isolated in this study may be a good replacement for those lacking vitamin B12 producing lactobacilli. We also showed that the LAB strains in this population were highly heterogeneous as determined by phenotypic and genotypic tests. After further evaluation in in vivo models and human clinical trials for determination of beneficial effects of our isolates in natural condition, incorporation of these potentially probiotic isolates in food is highly advantageous for Iranian populations.

## Supporting Information

S1 Fig Plasmid profiling of four isolated *Lactobacillus* based on plasmid extraction procedure. Lane 1: *L*. *plantarum* 13, lane 2: *L*. *brevis* 205, lane 3: 100bp ladder, lane 4: *L*. *plantarum* 240 and lane 5: *L*. *brevis* 470.
(TIF)

S1 Table Data set underlying the findings in our study in the manuscript. (XLSX)

S2 Table Statistical analysis of DAI and colon length in mice with acute DSS colitis with and without administration of potentially probiotic *Lactobacillus sp*. isolates. (DOCX)

## Abbreviations

LAB: Lactic acid bacteria
IBD: Inflammatory bowel disease
GIT: Gastrointestinal tract
UTI: Urinary tract infections
CTs: Common types
STs: Single types
WHO: World Health Organization
EAEC: EnteroAggregative*Escherichia coli*
ETEC: Entero Toxigenic *Escherichia coli*
EPEC: Entero Pathogenic*Escherichia coli*
DAI: Disease activity index
MPO: Myeloperoxidase
DSS: Dextran sulfatesodium
PBS: Phosphatebuffered saline
MRS: Man, Rogosa and Sharpe
PhPlate: Phene Plate system
ID: Identification level
RAPD: Random Amplified Polymorphic DNA
CLSI: Clinical and laboratory standards institute
MIC: Minimum inhibitory concentration
MOI: Multiplicity of infection
