# Supplementary figures and images for: Highly Heterogeneous Probiotic Lactobacillus Species in Healthy Iranians with Low Functional Activities
Source: PLoS One. 2015 Dec 8;10(12):e0144467. doi: 10.1371/journal.pone.0144467 (PMC4672925; doi:10.1371/journal.pone.0144467)

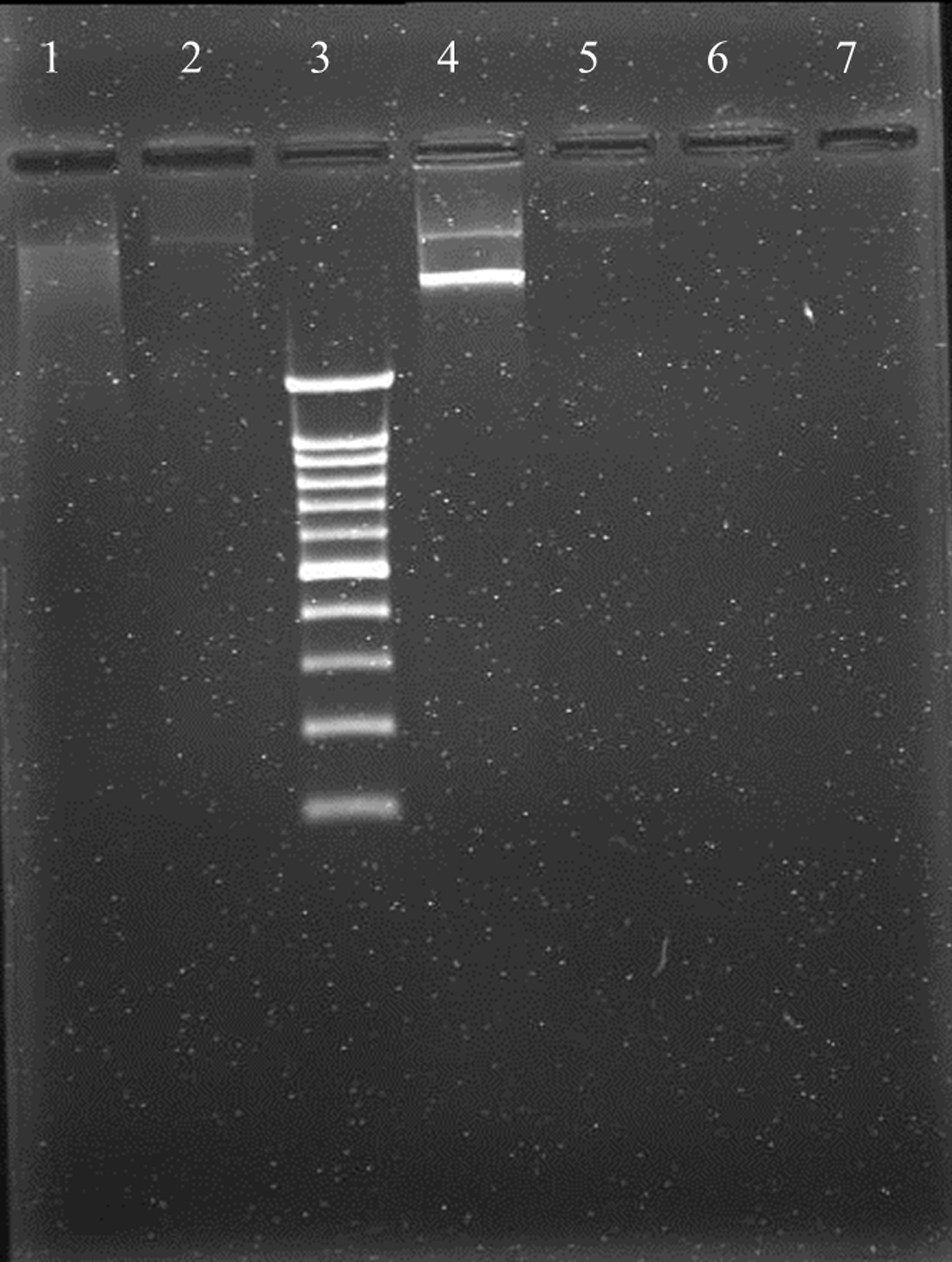

Supplement: S1 Fig — Lane 1: L. plantarum 13, lane 2: L. brevis 205, lane 3: 100bp ladder, lane 4: L. plantarum 240 and lane 5: L. brevis 470. (TIF) [file pone.0144467.s001.tif]
