# Supplementary material for: Highly Heterogeneous Probiotic Lactobacillus Species in Healthy Iranians with Low Functional Activities
Source: PLoS One. 2015 Dec 8;10(12):e0144467. doi: 10.1371/journal.pone.0144467 (PMC4672925; doi:10.1371/journal.pone.0144467)
Supplement: S2 Table — (DOCX) [file pone.0144467.s003.docx]

Descriptive table of mean and standard error of DAI in 12 mice group with different treatment

| **Descriptives** | | | | | | | | |
| --- | --- | --- | --- | --- | --- | --- | --- | --- |
| DAI |  |  |  |  |  |  |  |  |
|  | N | Mean | Std. Deviation | Std. Error | 95% Confidence Interval for Mean | | Minimum | Maximum |
|  |  |  |  |  | Lower Bound | Upper Bound |  |  |
| 1 | 5 | .0000 | .00000 | .00000 | .0000 | .0000 | .00 | .00 |
| 2 | 5 | 1.6667 | .40825 | .18257 | 1.1598 | 2.1736 | 1.00 | 2.00 |
| 3 | 5 | .1333 | .18257 | .08165 | -.0934 | .3600 | .00 | .33 |
| 4 | 5 | .3333 | .47140 | .21082 | -.2520 | .9187 | .00 | 1.00 |
| 5 | 5 | .1333 | .29814 | .13333 | -.2369 | .5035 | .00 | .67 |
| 6 | 5 | .8667 | .80277 | .35901 | -.1301 | 1.8634 | .00 | 2.00 |
| 7 | 5 | 1.5333 | .90062 | .40277 | .4151 | 2.6516 | .00 | 2.33 |
| 8 | 5 | 2.0667 | .64118 | .28674 | 1.2705 | 2.8628 | 1.33 | 3.00 |
| 9 | 5 | 1.6667 | .23570 | .10541 | 1.3740 | 1.9593 | 1.33 | 2.00 |
| 10 | 5 | 1.5333 | .38006 | .16997 | 1.0614 | 2.0052 | 1.00 | 2.00 |
| 11 | 5 | 1.0667 | .27889 | .12472 | .7204 | 1.4130 | .67 | 1.33 |
| 12 | 5 | 1.1333 | .18257 | .08165 | .9066 | 1.3600 | 1.00 | 1.33 |
| Total | 60 | 1.0111 | .80947 | .10450 | .8020 | 1.2202 | .00 | 3.00 |

Statistically analysis of DAI in 12 mice group with different treatment

| **Multiple Comparisons** | | | | | | |
| --- | --- | --- | --- | --- | --- | --- |
| DAI  LSD |  |  |  |  |  |  |
| (I) V1 | (J) V1 | Mean Difference (I-J) | Std. Error | Sig. | 95% Confidence Interval | |
|  |  |  |  |  | Lower Bound | Upper Bound |
| 1 | 2 | -1.66667^*^ | .29938 | .000 | -2.2686 | -1.0647 |
|  | 3 | -.13333 | .29938 | .658 | -.7353 | .4686 |
|  | 4 | -.33333 | .29938 | .271 | -.9353 | .2686 |
|  | 5 | -.13333 | .29938 | .658 | -.7353 | .4686 |
|  | 6 | -.86667^*^ | .29938 | .006 | -1.4686 | -.2647 |
|  | 7 | -1.53333^*^ | .29938 | .000 | -2.1353 | -.9314 |
|  | 8 | -2.06667^*^ | .29938 | .000 | -2.6686 | -1.4647 |
|  | 9 | -1.66667^*^ | .29938 | .000 | -2.2686 | -1.0647 |
|  | 10 | -1.53333^*^ | .29938 | .000 | -2.1353 | -.9314 |
|  | 11 | -1.06667^*^ | .29938 | .001 | -1.6686 | -.4647 |
|  | 12 | -1.13333^*^ | .29938 | .000 | -1.7353 | -.5314 |
| 2 | 1 | 1.66667^*^ | .29938 | .000 | 1.0647 | 2.2686 |
|  | 3 | 1.53333^*^ | .29938 | .000 | .9314 | 2.1353 |
|  | 4 | 1.33333^*^ | .29938 | .000 | .7314 | 1.9353 |
|  | 5 | 1.53333^*^ | .29938 | .000 | .9314 | 2.1353 |
|  | 6 | .80000^*^ | .29938 | .010 | .1981 | 1.4019 |
|  | 7 | .13333 | .29938 | .658 | -.4686 | .7353 |
|  | 8 | -.40000 | .29938 | .188 | -1.0019 | .2019 |
|  | 9 | .00000 | .29938 | 1.000 | -.6019 | .6019 |
|  | 10 | .13333 | .29938 | .658 | -.4686 | .7353 |
|  | 11 | .60000 | .29938 | .051 | -.0019 | 1.2019 |
|  | 12 | .53333 | .29938 | .081 | -.0686 | 1.1353 |
| 3 | 1 | .13333 | .29938 | .658 | -.4686 | .7353 |
|  | 2 | -1.53333^*^ | .29938 | .000 | -2.1353 | -.9314 |
|  | 4 | -.20000 | .29938 | .507 | -.8019 | .4019 |
|  | 5 | .00000 | .29938 | 1.000 | -.6019 | .6019 |
|  | 6 | -.73333^*^ | .29938 | .018 | -1.3353 | -.1314 |
|  | 7 | -1.40000^*^ | .29938 | .000 | -2.0019 | -.7981 |
|  | 8 | -1.93333^*^ | .29938 | .000 | -2.5353 | -1.3314 |
|  | 9 | -1.53333^*^ | .29938 | .000 | -2.1353 | -.9314 |
|  | 10 | -1.40000^*^ | .29938 | .000 | -2.0019 | -.7981 |
|  | 11 | -.93333^*^ | .29938 | .003 | -1.5353 | -.3314 |
|  | 12 | -1.00000^*^ | .29938 | .002 | -1.6019 | -.3981 |
| 4 | 1 | .33333 | .29938 | .271 | -.2686 | .9353 |
|  | 2 | -1.33333^*^ | .29938 | .000 | -1.9353 | -.7314 |
|  | 3 | .20000 | .29938 | .507 | -.4019 | .8019 |
|  | 5 | .20000 | .29938 | .507 | -.4019 | .8019 |
|  | 6 | -.53333 | .29938 | .081 | -1.1353 | .0686 |
|  | 7 | -1.20000^*^ | .29938 | .000 | -1.8019 | -.5981 |
|  | 8 | -1.73333^*^ | .29938 | .000 | -2.3353 | -1.1314 |
|  | 9 | -1.33333^*^ | .29938 | .000 | -1.9353 | -.7314 |
|  | 10 | -1.20000^*^ | .29938 | .000 | -1.8019 | -.5981 |
|  | 11 | -.73333^*^ | .29938 | .018 | -1.3353 | -.1314 |
|  | 12 | -.80000^*^ | .29938 | .010 | -1.4019 | -.1981 |
| 5 | 1 | .13333 | .29938 | .658 | -.4686 | .7353 |
|  | 2 | -1.53333^*^ | .29938 | .000 | -2.1353 | -.9314 |
|  | 3 | .00000 | .29938 | 1.000 | -.6019 | .6019 |
|  | 4 | -.20000 | .29938 | .507 | -.8019 | .4019 |
|  | 6 | -.73333^*^ | .29938 | .018 | -1.3353 | -.1314 |
|  | 7 | -1.40000^*^ | .29938 | .000 | -2.0019 | -.7981 |
|  | 8 | -1.93333^*^ | .29938 | .000 | -2.5353 | -1.3314 |
|  | 9 | -1.53333^*^ | .29938 | .000 | -2.1353 | -.9314 |
|  | 10 | -1.40000^*^ | .29938 | .000 | -2.0019 | -.7981 |
|  | 11 | -.93333^*^ | .29938 | .003 | -1.5353 | -.3314 |
|  | 12 | -1.00000^*^ | .29938 | .002 | -1.6019 | -.3981 |
| 6 | 1 | .86667^*^ | .29938 | .006 | .2647 | 1.4686 |
|  | 2 | -.80000^*^ | .29938 | .010 | -1.4019 | -.1981 |
|  | 3 | .73333^*^ | .29938 | .018 | .1314 | 1.3353 |
|  | 4 | .53333 | .29938 | .081 | -.0686 | 1.1353 |
|  | 5 | .73333^*^ | .29938 | .018 | .1314 | 1.3353 |
|  | 7 | -.66667^*^ | .29938 | .031 | -1.2686 | -.0647 |
|  | 8 | -1.20000^*^ | .29938 | .000 | -1.8019 | -.5981 |
|  | 9 | -.80000^*^ | .29938 | .010 | -1.4019 | -.1981 |
|  | 10 | -.66667^*^ | .29938 | .031 | -1.2686 | -.0647 |
|  | 11 | -.20000 | .29938 | .507 | -.8019 | .4019 |
|  | 12 | -.26667 | .29938 | .378 | -.8686 | .3353 |
| 7 | 1 | 1.53333^*^ | .29938 | .000 | .9314 | 2.1353 |
|  | 2 | -.13333 | .29938 | .658 | -.7353 | .4686 |
|  | 3 | 1.40000^*^ | .29938 | .000 | .7981 | 2.0019 |
|  | 4 | 1.20000^*^ | .29938 | .000 | .5981 | 1.8019 |
|  | 5 | 1.40000^*^ | .29938 | .000 | .7981 | 2.0019 |
|  | 6 | .66667^*^ | .29938 | .031 | .0647 | 1.2686 |
|  | 8 | -.53333 | .29938 | .081 | -1.1353 | .0686 |
|  | 9 | -.13333 | .29938 | .658 | -.7353 | .4686 |
|  | 10 | .00000 | .29938 | 1.000 | -.6019 | .6019 |
|  | 11 | .46667 | .29938 | .126 | -.1353 | 1.0686 |
|  | 12 | .40000 | .29938 | .188 | -.2019 | 1.0019 |
| 8 | 1 | 2.06667^*^ | .29938 | .000 | 1.4647 | 2.6686 |
|  | 2 | .40000 | .29938 | .188 | -.2019 | 1.0019 |
|  | 3 | 1.93333^*^ | .29938 | .000 | 1.3314 | 2.5353 |
|  | 4 | 1.73333^*^ | .29938 | .000 | 1.1314 | 2.3353 |
|  | 5 | 1.93333^*^ | .29938 | .000 | 1.3314 | 2.5353 |
|  | 6 | 1.20000^*^ | .29938 | .000 | .5981 | 1.8019 |
|  | 7 | .53333 | .29938 | .081 | -.0686 | 1.1353 |
|  | 9 | .40000 | .29938 | .188 | -.2019 | 1.0019 |
|  | 10 | .53333 | .29938 | .081 | -.0686 | 1.1353 |
|  | 11 | 1.00000^*^ | .29938 | .002 | .3981 | 1.6019 |
|  | 12 | .93333^*^ | .29938 | .003 | .3314 | 1.5353 |
| 9 | 1 | 1.66667^*^ | .29938 | .000 | 1.0647 | 2.2686 |
|  | 2 | .00000 | .29938 | 1.000 | -.6019 | .6019 |
|  | 3 | 1.53333^*^ | .29938 | .000 | .9314 | 2.1353 |
|  | 4 | 1.33333^*^ | .29938 | .000 | .7314 | 1.9353 |
|  | 5 | 1.53333^*^ | .29938 | .000 | .9314 | 2.1353 |
|  | 6 | .80000^*^ | .29938 | .010 | .1981 | 1.4019 |
|  | 7 | .13333 | .29938 | .658 | -.4686 | .7353 |
|  | 8 | -.40000 | .29938 | .188 | -1.0019 | .2019 |
|  | 10 | .13333 | .29938 | .658 | -.4686 | .7353 |
|  | 11 | .60000 | .29938 | .051 | -.0019 | 1.2019 |
|  | 12 | .53333 | .29938 | .081 | -.0686 | 1.1353 |
| 10 | 1 | 1.53333^*^ | .29938 | .000 | .9314 | 2.1353 |
|  | 2 | -.13333 | .29938 | .658 | -.7353 | .4686 |
|  | 3 | 1.40000^*^ | .29938 | .000 | .7981 | 2.0019 |
|  | 4 | 1.20000^*^ | .29938 | .000 | .5981 | 1.8019 |
|  | 5 | 1.40000^*^ | .29938 | .000 | .7981 | 2.0019 |
|  | 6 | .66667^*^ | .29938 | .031 | .0647 | 1.2686 |
|  | 7 | .00000 | .29938 | 1.000 | -.6019 | .6019 |
|  | 8 | -.53333 | .29938 | .081 | -1.1353 | .0686 |
|  | 9 | -.13333 | .29938 | .658 | -.7353 | .4686 |
|  | 11 | .46667 | .29938 | .126 | -.1353 | 1.0686 |
|  | 12 | .40000 | .29938 | .188 | -.2019 | 1.0019 |
| 11 | 1 | 1.06667^*^ | .29938 | .001 | .4647 | 1.6686 |
|  | 2 | -.60000 | .29938 | .051 | -1.2019 | .0019 |
|  | 3 | .93333^*^ | .29938 | .003 | .3314 | 1.5353 |
|  | 4 | .73333^*^ | .29938 | .018 | .1314 | 1.3353 |
|  | 5 | .93333^*^ | .29938 | .003 | .3314 | 1.5353 |
|  | 6 | .20000 | .29938 | .507 | -.4019 | .8019 |
|  | 7 | -.46667 | .29938 | .126 | -1.0686 | .1353 |
|  | 8 | -1.00000^*^ | .29938 | .002 | -1.6019 | -.3981 |
|  | 9 | -.60000 | .29938 | .051 | -1.2019 | .0019 |
|  | 10 | -.46667 | .29938 | .126 | -1.0686 | .1353 |
|  | 12 | -.06667 | .29938 | .825 | -.6686 | .5353 |
| 12 | 1 | 1.13333^*^ | .29938 | .000 | .5314 | 1.7353 |
|  | 2 | -.53333 | .29938 | .081 | -1.1353 | .0686 |
|  | 3 | 1.00000^*^ | .29938 | .002 | .3981 | 1.6019 |
|  | 4 | .80000^*^ | .29938 | .010 | .1981 | 1.4019 |
|  | 5 | 1.00000^*^ | .29938 | .002 | .3981 | 1.6019 |
|  | 6 | .26667 | .29938 | .378 | -.3353 | .8686 |
|  | 7 | -.40000 | .29938 | .188 | -1.0019 | .2019 |
|  | 8 | -.93333^*^ | .29938 | .003 | -1.5353 | -.3314 |
|  | 9 | -.53333 | .29938 | .081 | -1.1353 | .0686 |
|  | 10 | -.40000 | .29938 | .188 | -1.0019 | .2019 |
|  | 11 | .06667 | .29938 | .825 | -.5353 | .6686 |
| *. The mean difference is significant at the 0.05 level. | | | | | |  |

Colon Length Analysis

| **Descriptives** | | | | | | | | |
| --- | --- | --- | --- | --- | --- | --- | --- | --- |
| gut |  |  |  |  |  |  |  |  |
|  | N | Mean | Std. Deviation | Std. Error | 95% Confidence Interval for Mean | | Minimum | Maximum |
|  |  |  |  |  | Lower Bound | Upper Bound |  |  |
| 1 | 5 | 9.60 | 1.140 | .510 | 8.18 | 11.02 | 8 | 11 |
| 2 | 5 | 7.10 | 1.140 | .510 | 5.68 | 8.52 | 6 | 8 |
| 3 | 5 | 9.40 | 1.194 | .534 | 7.92 | 10.88 | 8 | 11 |
| 4 | 5 | 9.60 | .418 | .187 | 9.08 | 10.12 | 9 | 10 |
| 5 | 5 | 10.20 | .570 | .255 | 9.49 | 10.91 | 10 | 11 |
| 6 | 5 | 10.30 | .274 | .122 | 9.96 | 10.64 | 10 | 10 |
| 7 | 5 | 8.90 | .894 | .400 | 7.79 | 10.01 | 8 | 10 |
| 8 | 5 | 8.50 | .707 | .316 | 7.62 | 9.38 | 8 | 9 |
| 9 | 5 | 9.20 | .570 | .255 | 8.49 | 9.91 | 8 | 10 |
| 10 | 5 | 8.00 | 1.275 | .570 | 6.42 | 9.58 | 7 | 10 |
| 11 | 5 | 11.10 | .418 | .187 | 10.58 | 11.62 | 10 | 12 |
| 12 | 5 | 9.70 | .975 | .436 | 8.49 | 10.91 | 8 | 10 |
| Total | 60 | 9.30 | 1.299 | .168 | 8.96 | 9.64 | 6 | 12 |

Descriptive table of mean and standard error of colon length in 12 mice group with different treatment

| **Multiple Comparisons** | | | | | | |
| --- | --- | --- | --- | --- | --- | --- |
| gut  LSD |  |  |  |  |  |  |
| (I) V1 | (J) V1 | Mean Difference (I-J) | Std. Error | Sig. | 95% Confidence Interval | |
|  |  |  |  |  | Lower Bound | Upper Bound |
| 1 | 2 | 2.500^*^ | .547 | .000 | 1.40 | 3.60 |
|  | 3 | .200 | .547 | .716 | -.90 | 1.30 |
|  | 4 | .000 | .547 | 1.000 | -1.10 | 1.10 |
|  | 5 | -.600 | .547 | .278 | -1.70 | .50 |
|  | 6 | -.700 | .547 | .207 | -1.80 | .40 |
|  | 7 | .700 | .547 | .207 | -.40 | 1.80 |
|  | 8 | 1.100^*^ | .547 | .050 | .00 | 2.20 |
|  | 9 | .400 | .547 | .468 | -.70 | 1.50 |
|  | 10 | 1.600^*^ | .547 | .005 | .50 | 2.70 |
|  | 11 | -1.500^*^ | .547 | .009 | -2.60 | -.40 |
|  | 12 | -.100 | .547 | .856 | -1.20 | 1.00 |
| 2 | 1 | -2.500^*^ | .547 | .000 | -3.60 | -1.40 |
|  | 3 | -2.300^*^ | .547 | .000 | -3.40 | -1.20 |
|  | 4 | -2.500^*^ | .547 | .000 | -3.60 | -1.40 |
|  | 5 | -3.100^*^ | .547 | .000 | -4.20 | -2.00 |
|  | 6 | -3.200^*^ | .547 | .000 | -4.30 | -2.10 |
|  | 7 | -1.800^*^ | .547 | .002 | -2.90 | -.70 |
|  | 8 | -1.400^*^ | .547 | .014 | -2.50 | -.30 |
|  | 9 | -2.100^*^ | .547 | .000 | -3.20 | -1.00 |
|  | 10 | -.900 | .547 | .106 | -2.00 | .20 |
|  | 11 | -4.000^*^ | .547 | .000 | -5.10 | -2.90 |
|  | 12 | -2.600^*^ | .547 | .000 | -3.70 | -1.50 |
| 3 | 1 | -.200 | .547 | .716 | -1.30 | .90 |
|  | 2 | 2.300^*^ | .547 | .000 | 1.20 | 3.40 |
|  | 4 | -.200 | .547 | .716 | -1.30 | .90 |
|  | 5 | -.800 | .547 | .150 | -1.90 | .30 |
|  | 6 | -.900 | .547 | .106 | -2.00 | .20 |
|  | 7 | .500 | .547 | .365 | -.60 | 1.60 |
|  | 8 | .900 | .547 | .106 | -.20 | 2.00 |
|  | 9 | .200 | .547 | .716 | -.90 | 1.30 |
|  | 10 | 1.400^*^ | .547 | .014 | .30 | 2.50 |
|  | 11 | -1.700^*^ | .547 | .003 | -2.80 | -.60 |
|  | 12 | -.300 | .547 | .586 | -1.40 | .80 |
| 4 | 1 | .000 | .547 | 1.000 | -1.10 | 1.10 |
|  | 2 | 2.500^*^ | .547 | .000 | 1.40 | 3.60 |
|  | 3 | .200 | .547 | .716 | -.90 | 1.30 |
|  | 5 | -.600 | .547 | .278 | -1.70 | .50 |
|  | 6 | -.700 | .547 | .207 | -1.80 | .40 |
|  | 7 | .700 | .547 | .207 | -.40 | 1.80 |
|  | 8 | 1.100^*^ | .547 | .050 | .00 | 2.20 |
|  | 9 | .400 | .547 | .468 | -.70 | 1.50 |
|  | 10 | 1.600^*^ | .547 | .005 | .50 | 2.70 |
|  | 11 | -1.500^*^ | .547 | .009 | -2.60 | -.40 |
|  | 12 | -.100 | .547 | .856 | -1.20 | 1.00 |
| 5 | 1 | .600 | .547 | .278 | -.50 | 1.70 |
|  | 2 | 3.100^*^ | .547 | .000 | 2.00 | 4.20 |
|  | 3 | .800 | .547 | .150 | -.30 | 1.90 |
|  | 4 | .600 | .547 | .278 | -.50 | 1.70 |
|  | 6 | -.100 | .547 | .856 | -1.20 | 1.00 |
|  | 7 | 1.300^*^ | .547 | .022 | .20 | 2.40 |
|  | 8 | 1.700^*^ | .547 | .003 | .60 | 2.80 |
|  | 9 | 1.000 | .547 | .074 | -.10 | 2.10 |
|  | 10 | 2.200^*^ | .547 | .000 | 1.10 | 3.30 |
|  | 11 | -.900 | .547 | .106 | -2.00 | .20 |
|  | 12 | .500 | .547 | .365 | -.60 | 1.60 |
| 6 | 1 | .700 | .547 | .207 | -.40 | 1.80 |
|  | 2 | 3.200^*^ | .547 | .000 | 2.10 | 4.30 |
|  | 3 | .900 | .547 | .106 | -.20 | 2.00 |
|  | 4 | .700 | .547 | .207 | -.40 | 1.80 |
|  | 5 | .100 | .547 | .856 | -1.00 | 1.20 |
|  | 7 | 1.400^*^ | .547 | .014 | .30 | 2.50 |
|  | 8 | 1.800^*^ | .547 | .002 | .70 | 2.90 |
|  | 9 | 1.100^*^ | .547 | .050 | .00 | 2.20 |
|  | 10 | 2.300^*^ | .547 | .000 | 1.20 | 3.40 |
|  | 11 | -.800 | .547 | .150 | -1.90 | .30 |
|  | 12 | .600 | .547 | .278 | -.50 | 1.70 |
| 7 | 1 | -.700 | .547 | .207 | -1.80 | .40 |
|  | 2 | 1.800^*^ | .547 | .002 | .70 | 2.90 |
|  | 3 | -.500 | .547 | .365 | -1.60 | .60 |
|  | 4 | -.700 | .547 | .207 | -1.80 | .40 |
|  | 5 | -1.300^*^ | .547 | .022 | -2.40 | -.20 |
|  | 6 | -1.400^*^ | .547 | .014 | -2.50 | -.30 |
|  | 8 | .400 | .547 | .468 | -.70 | 1.50 |
|  | 9 | -.300 | .547 | .586 | -1.40 | .80 |
|  | 10 | .900 | .547 | .106 | -.20 | 2.00 |
|  | 11 | -2.200^*^ | .547 | .000 | -3.30 | -1.10 |
|  | 12 | -.800 | .547 | .150 | -1.90 | .30 |
| 8 | 1 | -1.100^*^ | .547 | .050 | -2.20 | .00 |
|  | 2 | 1.400^*^ | .547 | .014 | .30 | 2.50 |
|  | 3 | -.900 | .547 | .106 | -2.00 | .20 |
|  | 4 | -1.100^*^ | .547 | .050 | -2.20 | .00 |
|  | 5 | -1.700^*^ | .547 | .003 | -2.80 | -.60 |
|  | 6 | -1.800^*^ | .547 | .002 | -2.90 | -.70 |
|  | 7 | -.400 | .547 | .468 | -1.50 | .70 |
|  | 9 | -.700 | .547 | .207 | -1.80 | .40 |
|  | 10 | .500 | .547 | .365 | -.60 | 1.60 |
|  | 11 | -2.600^*^ | .547 | .000 | -3.70 | -1.50 |
|  | 12 | -1.200^*^ | .547 | .033 | -2.30 | -.10 |
| 9 | 1 | -.400 | .547 | .468 | -1.50 | .70 |
|  | 2 | 2.100^*^ | .547 | .000 | 1.00 | 3.20 |
|  | 3 | -.200 | .547 | .716 | -1.30 | .90 |
|  | 4 | -.400 | .547 | .468 | -1.50 | .70 |
|  | 5 | -1.000 | .547 | .074 | -2.10 | .10 |
|  | 6 | -1.100^*^ | .547 | .050 | -2.20 | .00 |
|  | 7 | .300 | .547 | .586 | -.80 | 1.40 |
|  | 8 | .700 | .547 | .207 | -.40 | 1.80 |
|  | 10 | 1.200^*^ | .547 | .033 | .10 | 2.30 |
|  | 11 | -1.900^*^ | .547 | .001 | -3.00 | -.80 |
|  | 12 | -.500 | .547 | .365 | -1.60 | .60 |
| 10 | 1 | -1.600^*^ | .547 | .005 | -2.70 | -.50 |
|  | 2 | .900 | .547 | .106 | -.20 | 2.00 |
|  | 3 | -1.400^*^ | .547 | .014 | -2.50 | -.30 |
|  | 4 | -1.600^*^ | .547 | .005 | -2.70 | -.50 |
|  | 5 | -2.200^*^ | .547 | .000 | -3.30 | -1.10 |
|  | 6 | -2.300^*^ | .547 | .000 | -3.40 | -1.20 |
|  | 7 | -.900 | .547 | .106 | -2.00 | .20 |
|  | 8 | -.500 | .547 | .365 | -1.60 | .60 |
|  | 9 | -1.200^*^ | .547 | .033 | -2.30 | -.10 |
|  | 11 | -3.100^*^ | .547 | .000 | -4.20 | -2.00 |
|  | 12 | -1.700^*^ | .547 | .003 | -2.80 | -.60 |
| 11 | 1 | 1.500^*^ | .547 | .009 | .40 | 2.60 |
|  | 2 | 4.000^*^ | .547 | .000 | 2.90 | 5.10 |
|  | 3 | 1.700^*^ | .547 | .003 | .60 | 2.80 |
|  | 4 | 1.500^*^ | .547 | .009 | .40 | 2.60 |
|  | 5 | .900 | .547 | .106 | -.20 | 2.00 |
|  | 6 | .800 | .547 | .150 | -.30 | 1.90 |
|  | 7 | 2.200^*^ | .547 | .000 | 1.10 | 3.30 |
|  | 8 | 2.600^*^ | .547 | .000 | 1.50 | 3.70 |
|  | 9 | 1.900^*^ | .547 | .001 | .80 | 3.00 |
|  | 10 | 3.100^*^ | .547 | .000 | 2.00 | 4.20 |
|  | 12 | 1.400^*^ | .547 | .014 | .30 | 2.50 |
| 12 | 1 | .100 | .547 | .856 | -1.00 | 1.20 |
|  | 2 | 2.600^*^ | .547 | .000 | 1.50 | 3.70 |
|  | 3 | .300 | .547 | .586 | -.80 | 1.40 |
|  | 4 | .100 | .547 | .856 | -1.00 | 1.20 |
|  | 5 | -.500 | .547 | .365 | -1.60 | .60 |
|  | 6 | -.600 | .547 | .278 | -1.70 | .50 |
|  | 7 | .800 | .547 | .150 | -.30 | 1.90 |
|  | 8 | 1.200^*^ | .547 | .033 | .10 | 2.30 |
|  | 9 | .500 | .547 | .365 | -.60 | 1.60 |
|  | 10 | 1.700^*^ | .547 | .003 | .60 | 2.80 |
|  | 11 | -1.400^*^ | .547 | .014 | -2.50 | -.30 |
| *. The mean difference is significant at the 0.05 level. | | | | | |  |
